# Difference in medical student performance in a standardized patient encounter between telemedicine and in-person environments

**DOI:** 10.1080/10872981.2024.2388422

**Published:** 2024-08-06

**Authors:** Emily M. Murphy, Ariella Stein, Reshma Pahwa, Maura McGuire, Tina Kumra

**Affiliations:** a Department of General Internal Medicine/Division of Hospital Medicine & Department of Pediatrics, Johns Hopkins University School of Medicine, Baltimore, MD, USA; b Department of General Internal Medicine, Johns Hopkins University School of Medicine, Baltimore, MD, USA; c Department of Physics, The STEM Academy in the Johns Hopkins University Applied Physics Lab, Laurel, MD, USA; d Department of Pediatrics, Johns Hopkins University School of Medicine, Baltimore, MD, USA

**Keywords:** Telemedicine, virtual, comparison, medical student, education, standardized patient

## Abstract

**Introduction:**

Telemedicine is an increasingly common form of healthcare delivery in the United States. It is unclear how there are differences in clinical performance in early learners between in-person and telemedicine encounters.

**Materials & Methods:**

The authors conducted a single-site retrospective cohort study of 241 second-year medical students to compare performance between in-person and telemedicine standardized patient (SP) encounters. One hundred and twenty medical students in the 2020 academic year participated in a telemedicine encounter, and 121 medical students in the 2022 academic year participated in an in-person encounter. SPs completed a multi-domain performance checklist following the encounter, and the authors performed statistical analyses to compare student performance between groups.

**Results:**

Students who completed in-person encounters had higher mean scores in overall performance (75.2 vs. 69.7, *p* < 0.001). They had higher scores in physical exam (83.3 vs. 50, *p* < 0.001) and interpersonal communication domains (95 vs. 85, *p* < 0.001) and lower scores in obtaining a history (73.3 vs. 80, *p* = 0.0025). There was no significant difference in assessment and plan scores (50 vs. 50, *p* = 0.96) or likelihood of appropriately promoting antibiotic stewardship (41.3% vs. 45.8%, *p* = 0.48).

**Conclusion:**

The authors identified significant differences in clinical performance between in-person and telemedicine SP encounters, indicating that educational needs may differ between clinical environments.

## Introduction

Telemedicine visits comprise a significant proportion of healthcare in the United States, with an average of 22.0% of adults reporting telehealth use in the prior 4 weeks between 2021 and 2022 [1]. While patient perception of telemedicine is positive [2–4], data on outcomes between in-person and telemedicine primary care encounters are heterogeneous [5,6]. Clinicians also report varying levels of comfort and satisfaction with telemedicine encounters [7–9]. Given the increasing need for clinicians who are competent in the delivery of telemedicine, the Association of American Medical Colleges (AAMC) has encouraged the growth and implementation of curricula to support telemedicine competencies for medical student learners [10,11].

However, it is unclear how best to educate medical students on the telemedicine encounter, and how these needs might differ from those of the in-person encounter. Clinician data reveal differences in physical exam (PE) technique and communication between these settings, indicating a need for foundational education [12,13]. Prior studies have demonstrated gaps in performance during trainee telemedicine encounters [14–16], suggesting that educational needs may differ depending on the clinical environment. As resources to provide telemedicine-specific curricula may be limited [17], an assessment of performance differences can aid in designing targeted interventions.

Standardized patient (SP) encounters permit medical trainees to practice clinical skills on live actors and have been shown to be an effective form of education and assessment [18]. SPs are often incorporated into Objective Structured Clinical Examinations (OSCEs), structured clinical assessments that include obtaining a history, performing a physical exam, and communicating findings and a plan [19]. Further, SPs have been integrated into telemedicine curricula for more than 20 years [20], with recent increases in the need to transition to telemedicine-based encounters in the setting of the COVID-19 pandemic [21].

The authors aimed to assess for differences in medical student performance in an SP-based OSCE between in-person and telemedicine environments. The primary outcome was overall student performance (total score) in the telemedicine encounter compared to performance in the in-person encounter. The authors secondarily assessed differences in student performance in encounter domains (history, PE, assessment and plan, and interpersonal communication skills, ICS) and differences in antibiotic stewardship.

## Materials & methods

The authors performed a retrospective cohort study of second-year medical students at Johns Hopkins University School of Medicine, who completed their Longitudinal Ambulatory Clerkship in December 2020 and December 2022. Primary and secondary outcomes, as above, were defined after the start of the data collection. To better understand differences in learning environments, the authors also performed a post-hoc analysis to compare longitudinal clinical experiences between the 2020 and 2022 cohorts.

One hundred and twenty medical students in the 2020 academic year participated in a telemedicine SP encounter and 121 students in the 2022 academic year participated in an in-person SP encounter. The study cohort did not include students who completed the clerkship in December 2021 as the SP case alternates every-other-year to minimize sharing of case information between classmates. Six weeks prior to the encounter, students had participated in a 4-h small group telemedicine role-play session. Pre-assessment clinical and telemedicine education otherwise included participation in a first-year clinical foundations of medicine course and experiential learning through longitudinal clinics.

The clerkship directors developed the SP case and performance checklist in collaboration with the Johns Hopkins Telemedicine Education Consortium, a group formed in March 2020 across multiple subspecialties to promote high telemedicine standards. The SP case, a patient presenting with persistent cough following influenza infection, was chosen as the content items were appropriate for in-person and telemedicine assessments. The patient has no red flags on history or PE suggestive of bacterial infection and PE is notable only for symptoms of an upper respiratory infection and dry cough, consistent with a diagnosis of post-viral cough. SPs received equivalent training for both telemedicine and in-person encounters: they received case materials before a virtual 4-h training session led by the same medicine simulation specialist. The case content and performance checklist items were reviewed in detail.

Student instructions were provided during a virtual 30-min orientation prior to the encounter. The chief complaint was provided on the door sheet prior to the start of the encounter. Following the encounter, SPs immediately completed the performance checklist for each student on the simulation center exam software.

Performance checklists included four major domains with discrete content items (Supplemental Table S1): patient history (15 items), PE (6 items), assessment and plan (4 items, including the antibiotic stewardship outcome), and ICS (4 items). The content items were first created by the clerkship directors, then reviewed and approved by three non-affiliated ambulatory physicians. The PE checklists between telemedicine and in-person sessions had the same number of content items but differed in content to account for expected differences in the telemedicine versus in-person PE. The checklists were otherwise identical. History, PE, and assessment and plan were scored as done correctly or not done correctly. ICS were scored on a 5-point Likert scale (strongly disagree, disagree, neutral, agree, strongly agree). The antibiotic stewardship secondary outcome was coded as correctly done if the student recommended against antibiotics. Scores were normalized to a percentage out of 100, and total scores were calculated as an average of domain scores.

The authors performed statistical analyses to assess their primary and secondary outcomes. Tests of normality were performed to determine the appropriate test. Performance domain scores were compared using Wilcoxon Rank Sum Test, and total performance scores were compared by t-test. Likert scale responses (non-dichotomized) for each ICS prompt were compared by Fisher’s exact test for reliability due to low cell counts. Likelihood of appropriately recommending against antibiotics was compared by chi-square test. Descriptive statistical analysis was done to describe frequency of correct performance of each domain item. Differences in clinical characteristics were compared by chi-square test, and differences in number of telemedicine sessions and total patients seen were compared by Wilcoxon Rank Sum Test. The authors used SAS Software, version 9.4 (SAS Inc, Cary, NC) for analysis. Two-tailed p-value <0.05 was considered significant. The project was acknowledged as exempt by the Johns Hopkins Medicine Institutional Review Board (IRB00270053).

## Results

There was no significant difference in clinic setting or specialty across clerkship years (Table 1). Students who completed the clerkship in 2020 had more telemedicine sessions (1, IQR 0–2, vs. 0, IQR 0–0, *p* < 0.001) and logged fewer total patients (35, IQR 34–38, vs. 51, IQR 50–53, *p* < 0.001) than students who completed the clerkship in 2022.

**Table 1.** Longitudinal ambulatory clinical experience of medical students cohorts participating in telemedicine and in-person standardized patient encounters.

	Telemedicine (*N* = 120)	In-person (*N* = 121)	p-value
**Clinic setting, n (%)**			0.5045^a^
Academic clinic	18 (15%)	25 (21%)	
Community clinic affiliated with academic center	43 (36%)	42 (35%)	
Community clinic unaffiliated with academic center	59 (49%)	54 (45%)	
**Clinic specialty, n (%)**			0.3218^a^
Internal medicine	58 (48%)	57 (47%)	
Internal medicine-Pediatrics/Family medicine	21 (18%)	14 (12%)	
Pediatrics	27 (23%)	27 (22%)	
Medical specialty	12 (10%)	22 (18%)	
Surgical specialty	2 (2%)	1 (1%)	
**Number of telemedicine sessions** Median (IQR)	1 (0-2)	0 (0-0)	**<0.001^b^**
**Total patients (telemedicine & in-person)** Median (IQR)	35 (34-38)	51 (50-53)	**<0.001**^**b**^

^a^Chi-Square Test.

^b^Wilcoxon Rank Sum Test.

Abbreviations: interquartile range, IQR.

Student performance varied across in-person and telemedicine SP encounters by domain, but students who participated in in-person encounters had a higher mean total score (75.2 vs. 69.7, *p* < 0.001; Table 2). Compared to students who participated in telemedicine encounters, students in in-person encounters had lower scores in obtaining a history (73.3 vs 80, *p* = 0.0025) but higher performance scores in the PE (83.3 vs. 50, *p* < 0.001) and ICS (95 vs. 85, *p* < 0.001). SP ratings of the students’ ICS behaviors were significantly different, and more SPs strongly agreed that the students exhibited the ICS behaviors during in-person encounters (Table 3). There was no significant difference in assessment/plan performance (50 vs. 50, *p* = 0.96), and there was no difference in promoting antibiotic stewardship through recommending against antibiotics (50 students, 41.3% vs. 55 students, 45.8%, *p* = 0.48).

**Table 2.** Differences in standardized patient performance scores between in-person and telemedicine encounters.

Performance domain	Scores (maximum 100)		p-value
	Telemedicine (*N* = 120)	In-person (*N* = 121)	
**History** Median (IQR)	80 (73.3 – 90)	73.3 (66.7 – 86.7)	**0.0025**^**a**^
**Physical Exam** Median (IQR)	50 (33.3 – 66.7)	83.3 (66.7 – 100)	**<0.001**^**a**^
**Assessment/Plan** Median (IQR)	50 (25 – 75)	50 (25 – 75)	0.96^a^
**Interpersonal Communication Skills** Median (IQR)	85 (80 – 100)	95 (90 – 100)	**<0.001**^**a**^
**Total** Mean (SD)	69.7 (10.9)	75.2 (10.5)	**<0.001**^**b**^

^a^Wilcoxon Rank Sum Test.

^b^T-test.

Abbreviations: interquartile range, IQR; standard deviation, SD.

**Table 3.** Differences in standardized patient evaluation of interpersonal communication skills between in-person and telemedicine encounters.

Interpersonal Communication Skill	Likert scale response	Telemedicine N (%)	In-person N (%)	p-value^a^
Student introduced themselves and explained their role	Disagree	1 (0.8)	1 (0.8)	**0.0427**
	Neutral	4 (3.3)	7 (5.8)	
	Agree	45 (37.5)	27 (22.3)	
	Strongly agree	70 (58.3)	86 (71.1)	
Student allowed me to explain my chief concern and other elements of the history without excessive verbal interruptions	Neutral	0 (0)	8 (6.6)	**<0.001**
	Agree	58 (48.3)	10 (8.3)	
	Strongly agree	62 (51.7)	103 (85.1)	
Student expressed emotional support and caring for me	Disagree	2 (1.7)	0 (0)	**<0.001**
	Neutral	6 (5.0)	19 (15.7)	
	Agree	65 (54.2)	24 (19.8)	
	Strongly agree	47 (39.2)	78 (64.5)	
Student used language I easily understand	Disagree	2 (1.7)	0 (0)	**<0.001**
	Neutral	4 (3.3)	9 (7.4)	
	Agree	65 (54.2)	17 (14.1)	
	Strongly agree	49 (40.8)	95 (78.5)	

^a^Fisher’s Exact Test.

## Discussion

The authors found that medical student performance in identical SP cases differed depending on whether the encounter was in-person or telemedicine. Students in in-person encounters received higher total performance scores due to higher performance on PE and ICS but scored lower on obtaining a history compared to students in telemedicine encounters.

Telemedicine PE performance was worse compared to in-person PE performance. Poor telemedicine PE performance has previously been noticed in both medical students [14,22] and residents and fellows [15,16]. While one study demonstrated improvement in PE performance after role-play or demonstration interventions, PE performance scores remained low [22]. In a scoping review of the virtual PE, most studies demonstrated that telemedicine and in-person PEs were equivalent (54 studies out of 61), but 7 studies concluded that the telemedicine PE was inferior, suggesting that gaps exist at the clinician level as well [13]. Future efforts to improve telemedicine outcomes in education should focus on teaching PE skills to ensure that trainees and providers across the spectrum of medical careers achieve the expected AAMC competencies [10]. Based on the authors’ findings, the Longitudinal Ambulatory Clerkship curriculum now includes asynchronous education on telemedicine-specific PE maneuvers.

The authors observed that student scores in ICS were also lower in telemedicine encounters than in-person encounters. Prior research suggests that this gap does not exist at the physician level: in a noninferiority clinical trial, patients randomized to telemedicine or virtual consults reported equal satisfaction with the physician’s interpersonal skills [23]. Interestingly, the same authors noted in a separate study that the physicians in telemedicine encounters had increased conversational dominance and that patients required more repetition [12], which the authors’ SPs similarly observed. It is unclear if the performance gap seen between medical students and physicians requires telemedicine-specific communication training, but as medical students will likely be exposed to telemedicine encounters in their training, they should receive increased support to feel comfortable with telemedicine communication. In a published telemedicine communication curriculum, students responded positively to the curriculum and reported an increased perceived comfort with conducting telemedicine visits, but the impact on the curriculum on patient perception of communication was not studied [24].

Although student performance in telemedicine encounters was overall worse than in-person encounters, students performed equally on the assessment and plan portion of the encounter. The primary plan outcome was whether or not the student appropriately recommended against antibiotics, and the authors observed no difference between groups. It is possible that students in the telemedicine group performed better on the history portion due to prioritizing the history in the context of a deprioritized PE, leading to similar final assessments. Student assessment and plan performance in both telephonic and video telemedicine encounters is low at baseline, with variable improvement after educational interventions [22,25], including improved cost-effective care [26]. Spaced exposure to telemedicine across clerkships and learning environments may be important for developing consistent clinical reasoning skills. The authors plan to incorporate telemedicine simulation into the medical school high-value care curriculum, which has been shown to decrease spending in an in-person SP encounter [27].

Limitations of this study include the fact that it was a retrospective study performed at a single site with a limited sample size. It is possible that curricular differences in preclinical education, specifically telemedicine education, may lead to different performance outcomes outside of our site. While the checklist was reviewed by unaffiliated physicians, it was not validated, and prior systematic reviews highlight that checklist heterogeneity may impact the ability to interpret student performance both within and between institutions [28]. Students who participated in the in-person SP encounter saw significantly more patients during their ambulatory clerkship, potentially indirectly improving clinical performance, and students in the telemedicine cohort participated in significantly more telemedicine sessions, potentially specifically improving telemedicine performance. While the variation in clinical exposure occurred in the unique context of the 2020 coronavirus pandemic, the differences in student performance across domains are consistent with those reported in previous studies [14,22,25]. Further research will be needed to analyze performance trends over time.

## Conclusions

In this retrospective cohort study of medical students participating in telemedicine or in-person SP encounters, the authors aimed primarily to compare overall performance between encounter type. They found that students had higher total performance scores during in-person encounters. However, student performance varied by domain and there was no difference in antibiotic stewardship between encounter type. Future research should aim to improve telemedicine competences longitudinally over medical training through targeted communication and PE interventions.

## Supplementary Material

TelemedicineManuscript_SupplementalTable_Final.docx
