# Supplementary material for: Difference in medical student performance in a standardized patient encounter between telemedicine and in-person environments
Source: Med Educ Online. 2024 Aug 6;29(1):2388422. doi: 10.1080/10872981.2024.2388422 (PMC11500675; doi:10.1080/10872981.2024.2388422)
Supplement: TelemedicineManuscript_SupplementalTable_Final.docx [file ZMEO_A_2388422_SM7701.docx]

Supplemental Table 1: Medical student performance by checklist item during telemedicine and in-person standardized patient encounters

| **Checklist item** | **Telemedicine performance (% performed correctly)**  **N=120** | **In-person performance**  **(% performed correctly)**  **N=121** |
| --- | --- | --- |
| **History** | | |
| Student asked about the onset of the cough. | 98 | 100 |
| Student asked about the frequency of the cough. | 89 | 73 |
| Student asked about quality of cough. | 95 | 91 |
| Student asked what makes the cough better or worse | 79 | 76 |
| Student asked about ear or throat pain. | 68 | 73 |
| Student asked about SOB or difficulty breathing. | 84 | 71 |
| Student asked about fevers or chills. | 73 | 96 |
| Student asked about history of asthma or COPD. | 58 | 20 |
| Student asked about sick contacts. | 68 | 51 |
| Student asked me about my medical history. | 94 | 87 |
| Student asked about drug, alcohol, tobacco use. | 79 | 95 |
| Student asked me if I wanted to stop smoking. | 45 | 67 |
| Student elicited at least one review of system related to chief complaint | 83 | 84 |
| Student asked about current medications. | 91 | 76 |
| Student asked about allergies. | 87 | 49 |
| **Physical exam** | | |
| ^a^Student looked in my ears with otoscope.  ^b^Student either asked me to turn my head from side to side to inspect my ears or asked me to pull down on my ears and asked me if I had pain with pulling | 25 | 60 |
| Student inspected my throat with a light | 74 | 89 |
| ^a^Student tapped on my sinuses and asked if that was uncomfortable  ^b^Student asks me to tap on my sinuses and asked if that was uncomfortable | 20 | 83 |
| ^a^Student listened to my lungs with stethoscope  ^b^Student asked me to remove sweater or jacket to assess my work of breathing | 43 | 98 |
| ^a^Student performed at least one: percussed my back; asked me to say “ee” while listening with stethoscope; or asked me to say “99” while feeling my back with both hands  ^b^Student asked me to take deep breath and exhale at least once) | 55 | 97 |
| ^a^Student checked my neck for swollen glands  ^b^Student asked me to palpate my neck for swollen glands | 63 | 74 |
| **Assessment and plan** | | |
| Student provided correct diagnosis (viral upper respiratory infection) | 50 | 58 |
| Student recommended against antibiotics | 46 | 41 |
| Student discussed follow-up plan | 61 | 40 |
| Student addressed smoking cessation | 39 | 57 |

^a^In-person encounter checklist item

^b^Telemedicine encounter checklist item
